# Readmissions to kidney transplantation department: incidence, causes and risk factors

**DOI:** 10.11604/pamj.2022.41.305.31067

**Published:** 2022-04-14

**Authors:** Syrine Tlili, Lilia Ben Fatma, Ikram Mami, Hiba Ghabi, Badreddine Kaab, Smaoui Wided, Madiha Krid, Soumaya Beji, Lamia Rais, Fethi Ben Hamida, Hela Jebali, Mohamed Karim Zouaghi

**Affiliations:** 1 Department of Nephrology, la Rabta Hospital, Jabbari, 1007, Tunis, Tunisia,; 2 Research Laboratory in Renal Pathology LR00SP01, Medicine School of Tunis, Tunis El Manar University, Tunis, Tunisia

**Keywords:** Risk factor, hospital readmission, kidney transplantation

## Abstract

**Introduction:**

hospital readmission after kidney transplantation is an important metric for health care quality, which associated with increased morbidity, costs and transition-of-care errors. It is influenced by population demographics and the comprehensiveness of the healthcare system. The aim of this study was to evaluate incidence causes and risk factors associated with hospital readmission within the first year after transplantation.

**Methods:**

all patients undergoing kidney transplantation at a single center over a ten-year period were analyzed via retrospective chart review. A multivariable logistic regression analysis was performed to identify associated factors.

**Results:**

in 86 patients, the incidence of unplanned readmissions within the first year was 68.6% (n = 59). The main reasons for HR were infection (33%), renal events (32%), surgical complications (16%), and metabolic disturbances (9%). In univariate analyses, hospital readmission was associated with Dyslipidemia p=0.04; OR=2.6; 95% CI= [1.93-13.17], anemia p=0.011; OR=4.5; 95% CI = [1.33-15.6], hemodialysis p=0,012; OR=4.8 ; 95% CI= [1.3-18.5], new onset diabetes after transplantation p=0.05 ; OR=3.5 ; 95% CI= [1.6-13,80], medical history of cardiomyopathy p=0,016 ; OR=6.4 ; 95% CI = [5.4-7.5]. While independent risk factors were: hemodialysis vintage and cardiomyopathy. There was no difference in one-year patient survival and death-censored graft survival in HR group and non-HR group.

**Conclusion:**

hospital readmissions severely affect a patient's physical and mental well-being after kidney transplantation, which is also independently associated with morbidity. Our study showed that risk factors associated with hospital readmission often reflect pretransplant comorbidity.

## Introduction

End-stage renal disease (ESRD) is a global scourge and a major health problem, which is increasing the burden on health systems. This is not only because of the onerous nature of its care but also because of its impact on professional and social life. Among the treatments for ESRD, kidney transplantation (KT) is now the replacement therapy of choice. It offers better results than dialysis, whether in terms of survival, quality of life, or financial cost [1]. It offers a new therapeutic era and revolutionizes the prognosis of chronic kidney disease, its cost and the quality of life of patients [2]. However, KT remains a therapy that is not devoid of complications and some of which require a hospital readmission. Since, hospital readmission (HR) has been associated with a two-fold increase in the risk of graft failure and an increase of 50 to 75% in patient mortality [3-5]. We aimed to identify the population at risk of HR in order to ameliorate the results of KT with an early screening and management of complications. The aims of our study were to determine the incidence, cause and risk factors of HR after KT during the first year after transplantation.

## Methods

**Study design and setting**: this was a nine-year retrospective cohort analysis carried out at the nephrology, dialysis and transplantation department of the Rabta Hospital Tunis, Tunisia.

**Study population**: all adult kidney transplant recipients (18 years and above) who received a kidney transplant between November 29, 2010 and November 29, 2019 from living and cadaveric donors.

**Data collection**: data were collected on consecutive adult kidney transplant recipients who received a kidney transplant between November 29, 2010 and November 29, 2019. It was performed using a database of kidney transplant recipients at the nephrology, dialysis and transplantation department of the Rabta Hospital Tunis, Tunisia.

**Study procedures**: the main study outcome was readmission up to one-year post discharge of kidney transplantation. Hospital readmission is defined as at least 1 readmission (planned readmission or acute causes) after discharge from the index hospitalization. All hospital readmissions within the first year following discharge were analyzed. In order to extract risk factors, data included recipient characteristics (age, sex, body mass index, comorbidities, dialysis need and access, cause of ESRD, CMV status), donor characteristics (living or cadaveric donor, panel reactive antibody level, HLA mismatches with donor, CMV status), transplant characteristics (cold ischemia time, warm ischemia time, induction agent, maintenance immunosuppression), and complications following kidney transplantation.

**Statistical analysis**: statistical analyses were performed using SPSS version 25.0. Descriptive statistics were used to report baseline patient characteristics. Student´s t-test, Mann- Whitney U test and Chi-square test were utilized to compare the differences between patients with or without readmission after KT. To find the potential risk factors, a multivariate analysis was applied based on a binary logistic regression adjusted for the variables significantly associated with the event studied and identified by the univariate analysis, as well as the variables described in the literature as potential confounding factors. The confidence interval is 95%, and P-value will be considered significant if it is equal to or less than 0.05.

## Results

**General characteristics**: a total of 86 consecutive KT were performed at our center during the study period. The mean age was 35.3 ±9.7 (17-58 years) and 57 patients (66%) were male. Primary renal disease was to glomerulonephritis in 44%. Overall, 60 patients (70%) had been on hemodialysis with a median time on dialysis before KT of 19 months (0-120 months). The majority of our patients had received a kidney from living donor in 95% of cases. A summary of baseline characteristics of patients are presented in Table 1.

**Table 1:** baseline patient´s characteristic

Variables	Readmission group(n=59)	Non readmission group(n= 27)	P-value
**Mean age (year)**	34.9	36.4	NS
**Gender**			
Male	40(68%)	17(63%)	NS
Female	19(32%)	10(37%)	
**Body mass index (kg/m^2^)**	24.2	26.3	NS
**Comorbidities**			
Hypertension	40(68%)	20(74%)	NS
Diabetes	3(5%)	4 (15%)	NS
Dyslipidemia	40(68%)	12(44%)	0.04
cardiomyopathy	25(42%)	10(37%)	0.05
**Dialysis method**			
Hemodialysis	36(61%)	24(89%)	0.012
Peritoneal dialysis	22(37%)	3(11%)	NS
**Anemia**	54(92%)	19(70%)	0.011
**Living donor**	56(95%)	26(96%)	NS
**Delayed graft function**	9(5%)	5(18%)	NS
**New onset diabetes**	18(31%)	3(11%)	0.016

**Incidence and causes of hospital readmission**: during the period of our study, 59 patients (70%) required at least one HR. We noted a total readmission number of 118 within the first year after KT, with an average of two episodes per patient. Overall, 25 patients had one readmission during the first year, 20 patients experienced two readmissions, eight patients had three admissions and six patients had more than three HR during the period of study. A total of 29 patients (52%) were readmitted within 30 days, 49 (83%) were readmitted within 6 months, and 15 patients (25%) within one year. In our sample, HR were unplanned ones in 70% of cases. The main cause was infectious episodes in 34% of cases followed by renal issues among 33% of patients and 15% were for surgical complications. The different causes are detailed in Table 2. The median HR duration was seven days.

**Table 2:** causes of hospital readmission within the first year

Causes of readmission	Percentage
**Infectious disease:**	34
Urinary tract infection	16
Lower lobe pneumonia	7
Cytomegalovirus infection	9
parvovirus B19 infection	1
**Renal events:**	33
Graft biopsy	19
Graft dysfunction or graft rejection	8
Acute graft injury	6
**Surgical complication:**	15
Ablation of peritoneal dialysis catheter	11
Osteonecrosis of the femoral head	2
Cellulitis	1
peritonitis	1
**Gastro-intestinal events:**	6
Diarrhea	5
Abdominal pain	1
**Metabolic disorder:**	8
New onset diabetes	6
Diabetic ketoacidosis	1
Hypercalcemia	1
**Other complications:**	5
Chest pain	1
Dyspnea	4

**Risk factors for hospital readmission**: on univariable analysis, risk factors for HR within the first year post transplantation were: dyslipidemia p=0,04; OR=2.6; 95% CI= [1,93-13,17], anemia p=0,011; OR=4.5; 95% CI = [1,3-15,6], hemodialysis p=0,012; OR=4.8; 95% CI= [1,3-18.5], new onset diabetes after transplantation p=0,05; OR=3.5; 95% CI= [1,6-13,80], medical history of cardiomyopathy p=0,016; OR=6.4; 95% CI = [5.4-7.5]. While Logistic regression analysis showed that only those with a medical history of cardiomyopathy (P=0,041; OR = 10.6, 95% CI= 3.5-52.8), and those who were on hemodialysis (P=0.04; OR= 8.746, 95% CI=2.72-15.2), were associated with an increased risk of HR in the first year after renal transplantation. There was no difference in one-year patient survival and death-censored graft survival in HR group and non-HR group.

## Discussion

As well as the increase in the rate of chronic renal disease and ESRD over the years, there was also a steadily increase in the rate of KT [6]. Thus, the number of complications who will require HR is likely to rise in step with the increase in the rate renal transplantation. Therefore, HR is an important metric for both clinicians and healthcare institutions to assess their medical practices and improve the abilities for health care. Out of 86 patients included in our analysis, 69% of them experienced at least one HR during the first year post KT. This result is in line with precedent results of other authors. Indeed, in a study conducted by Chu *et al*. which included 40461 patients, they reported that the rate of HR within the first year was of 62.9% [4]. In addition, Bergman *et al*. reported in their analysis, a rate of 59.2% of patients with at least one HR [7]. The variation in the rate of readmission among other centers may be owed to interactions between patient characteristics and patient care [8].

During our study, patients were readmitted mainly for infectious episodes, renal issues and surgical complications. Thoroughly revising precedent reports, post KT rehospitalization´s causes depend on the period of study [9]. In fact, we found that infectious complications are more frequent during the first six months [10-12], in particular during the first month post KT with a rate of 50% [13]. This may be explained by immunosuppression, which must be at its maximum in this period. In our sample, most common infectious complication were urinary tract and pulmonary infection which is in line with previous study [14,15]. It has been reported that graft dysfunction is responsible for 30 to 45% of HR in the first year [9,10], while this rate is only at 8% in our study. This is may be because a close follow up of our patients with frequent and preventive clinic visits. As for surgical causes, it used to be more frequent in the early post-operative period, such as lymphocele and urinary leaks and decrease thereafter [10,16]. According to the literature, hospital readmission is a strong indicator for graft loss and patient´s mortality [3]. Thus, identifying population at high risk of readmission seems to be imperative in order to prompt careful patient selection and follow-up for patients after their discharge.

Our statistical multivariable analysis has shown that hemodialysis before KT and a medical history of cardiomyopathy were associated with increased odds of rehospitalization within the first year. Our results are consistent with previous findings [3,4,15,17]. Indeed, it is not surprising to find that Risk factors associated with HR often reflect pretransplant comorbidities. Hemodialysis is known to increase morbidity and mortality among patients by several mechanisms. Hemodialysis is associated with rapid progression of cardiovascular changes, particularly left ventricular hypertrophy and vascular calcification [18]. Moreover, it has been reported to be associated with increased chronic inflammatory status, poor nutritional status and impaired immunological function [19]. Another important fact, investigators reported that long-term hemodialysis is associated with an alteration of the intestinal mucosa reducing the absorption of immunosuppressant and thus exposing the patient to a risk of rejection [20].

Other authors have, also reported the association between heart impairment and rehospitalization. In fact, Bergman *et al*. had reported that heart failure is not only a risk factor for first HR but also recurrent readmission [7]. This is probably due to a low baseline health status and a decrease in physiologic reserve which makes the patients more susceptible to decompensation in response to the least stress [7]. The consequence of such healthcare status would be an increase in the requirements for hospital readmission after discharge. There are several limitations of our study: it is a single-center analysis conducted among a small sample size. In addition, data was retrospectively collected from medical history of patients, so some specific details may be missed, which may reduce the power of our study.

## Conclusion

In conclusion, HR is a great metric that reflect patient´s health status, increase healthcare costs and specially a marker of both patient and graft morbidity and mortality. Most frequent causes of rehospitalization are infectious complication and surgical issues in the first months after KT, whose frequency decrease thereafter in favor of renal events. Our study showed that hemodialysis and a history of heart failure were highly associated with HR probability. Indeed, most of previous studies das shown that rehospitalization is associated with low baseline health status with several comorbidities and high frailty. That is why more studies are needed to identify specific risk factors for early and late HR in order to improve management, life quality and results of KT.

### What is known about this topic

- End-stage renal disease (ESRD) is a global scourge and a major health problem, which is increasing the burden on health systems;
- Hospital readmission (HR) after kidney transplantation is known to be associated with a two-fold increase in the risk of graft failure and an increase of 50 to 75% in patient mortality.

### What this study adds

- The main causes of hospital readmission during the first year after kidney transplantation;
- The risk factors of hospital readmission within transplant population.
